# Supplementary material for: A cross-sectional study of barriers to cervical cancer screening uptake in Ghana: An application of the health belief model
Source: PLoS One. 2020 Apr 30;15(4):e0231459. doi: 10.1371/journal.pone.0231459 (PMC7192489; doi:10.1371/journal.pone.0231459)
Supplement: S1 File — (DOCX) [file pone.0231459.s001.docx]

**APPENDIX**

**QUESTIONNAIRE**

This questionnaire is for academic research and designed to find out the barriers influencing cervical cancer screening uptake among women in Kenyasi, Ghana. Your responses will be treated confidential and all information will be reported as aggregated data. Hence, you are not required to write your name. There are no wrong or right answers. This is just to seek your opinion on the subject. Kindly tick the appropriate spaces provided or write what you think in the open-ended questions. I will be grateful if you can answer all questions to the best of your ability.

Thank you.

## SECTION A: SOCIODEMOGRAPHIC DATA

1. Age; …………………….
2. Occupation; ……………………………………
3. Educational background

Illiterate ( ) b. Primary ( ) c. JHS ( ) d. SHS ( ) e. Tertiary ( )

1. Religion

Christian ( ) b. Muslim ( ) c. Traditional ( ) d. Buddhism ( ) e. Others……………

1. Marital status

Married ( ) b. Single ( ) c. Divorced ( ) d. Widowed ( ) e. Informal union ()

1. Number of child birth…………………………
2. Family history of cervical cancer. a. Yes ( ) b. No ( )

## SECTION B: AWARENESS OF CERVICAL CANCER

1. Have you heard of cervical cancer? a. Yes ( ) b. No ( )
2. How did you first learn about cervical cancer?
3. Never heard of it before today ( ) e. From a family member ( )
4. From a friend ( ) f. From a doctor/ nurse ( )
5. From school personnel ( ) g. From radio, TV, magazine ( )
6. I don’t remember ( ) h. Other sources………………….
7. Cervical cancer is a sexually transmitted disease. a. Yes ( ) b. No ( ) c. I don’t know( )
8. Cervical cancer is preventable. a. Yes ( ) b. No ( ) c. I don’t know ( )
9. Cervical cancer is preventable through vaccination of young girls. a. Yes ( ) b. No ( ) c. I don’t know ( )
10. Cervical cancer is curable in hospitals when diagnosed early. a. Yes () b. No ( ) c. I don’t know ()
11. Please tick the risk factors of cervical cancer.

Early onset of sexual activity. ( )

Infection with a sexually transmitted germ/virus (HPV). ( )

Multiple male sexual partners. ( )

Smoking cigarettes/tobacco. ( )

Grand multiparity. ( )

1. Please tick the symptoms of cervical cancer.

Intermenstrual vaginal bleeding. ( )

Post-menopausal bleeding. ( )

Vaginal bleeding. ( )

Post-coital vaginal bleeding. ( )

Excessive vaginal discharge, often with offensive smell. ( )

Lower abdominal pain. ( )

Pain in the genital during sexual intercourse. ( )

1. Have you heard of cervical cancer screening? a. Yes ( ) b. No ( )
2. Have you been screened of cervical cancer before? a. Yes ( ) b. No ( )
3. If yes, when was the last time you had it? ……………………………………
4. How often do you go for screening?
5. Once a year ( ) c. Less often than every 5 years ( )
6. Every 2-5 years ( ) d. I don’t know ( )
7. Are you interested in participating in cervical screening? a. Yes ( ) b. No ( )

**SECTION C: PERCEIVED THREAT OF CERVICAL CANCER**

The following questions will prevent you from participating in screening of cervical cancer

1. Does the thought of cancer scare you? a. Yes ( ) b. No ( ) c. I don’t know ( )
2. Do you think you are susceptible to cervical cancer? a. Yes ( ) b. No ( ) c. I don’t know ()
3. Are you afraid of a bad diagnosis? a. Yes ( ) b. No ( ) c. I don’t know ( )

**SECTION D: PERCEIVED BENEFITS OF CERVICAL CANCER SCREENING**

1. Do you think cervical cancer screening is important? a. Yes ( ) b. No ( ) c. I don’t know( )
2. Do you believe cervical cancer can be cured? a. Yes ( ) b. No ( ) c. I don’t know ( )

**SECTION E: PERCEIVED BARRIERS OF CERVICAL CANCER SCREENING**

## Psychosocial barrier of cervical screening

The following questions will prevent you from participating in screening of cervical cancer:

1. Do you think cervical screening is painful? a. Yes ( ) b. No ( ) c. I don’t know ( )
2. Do you find cervical screening embarrassing? a. Yes ( ) b. No ( ) c. I don’t know ( )
3. Do you believe cervical cancer is a curse from the gods? a. Yes ( ) b. No ( ) c. I don’t know( )
4. Does your religion have anything against cervical screening? a. Yes () b. No () c. I don’t know ( )
5. Does your culture forbid cervical screening? a. Yes ( ) b. No ( ) c. I don’t know ( )

## Socioeconomic barriers to screening

The following questions will prevent you from participating in screening of cervical cancer:

1. Is cervical screening expensive? a. Yes ( ) b. No ( ) c. I don’t know ( )
2. If yes, how much can you afford?………………………………………..
3. Is the transport system to the health facility good? a. Yes ( ) b. No ( ) c. I don’t know ( )
4. Do you have time for cervical screening? a. Yes ( ) b. No ( ) c. I don’t know ( )

## Healthcare system barriers to screening

The following questions will prevent you from participating in screening of cervical cancer:

38. Do you know any health facility offering cervical screening services?

a. Yes ( ) b. No ( ) c. I don’t know ( )

39. Do you feel comfortable with a male health personnel rendering screening services?

a. Yes ( ) b. No ( ) c. I don’t know ( )

40. Are there long waiting time at the health facility?

a. Yes ( ) b. No ( ) c. I don’t know ( )

41. Is it difficult to communicate with health personnel?

a. Yes ( ) b. No ( ) c. I don’t know ( )

## SECTION F: CUES FOR ACTION

42. Do you think prioritizing early morning and late evening appointments for patient of working age increase screening uptake?

a. Yes ( ) b. No ( ) c. I don’t know ( )

43. Do you think ensuring that patient’s awareness of available facility for cervical screening improve behaviors of screening?

a. Yes ( ) b. No ( ) c. I don’t know ( )

44 Will seeking female health personnel to provide screening services increase screening uptake?

a. Yes ( ) b. No ( ) c. I don’t know ( )
