# Supplementary material for: A cross-sectional study of barriers to cervical cancer screening uptake in Ghana: An application of the health belief model
Source: PLoS One. 2020 Apr 30;15(4):e0231459. doi: 10.1371/journal.pone.0231459 (PMC7192489; doi:10.1371/journal.pone.0231459)
Supplement: Attachment — Submitted filename: summary of the report- Cervical Cancer.docx [file pone.0231459.s002.docx]

# A CROSS-SECTIONAL STUDY OF BARRIERS TO CERVICAL CANCER SCREENING UPTAKE IN GHANA: AN

# APPLICATION OF THE HEALTH BELIEF MODEL

# SUMMARISED COMMENTS

| **Section** | **Comment, question, suggestion.** |
| --- | --- |
| **Abstract** | 1. On the introduction report high incidence and high mortality of Cervical Cancer with low screening uptake (needs to quantify) 2. Study period is missing in methods section 3. Reports descriptive Cross-sectional study design while multivariable logistic regression was done ( needs to change to analytical cross-sectional study design) 4. Missed recommendations and policy implications on Conclusion. |
| **Introduction**  **Background** | 1. What is the burden of Cervical Cancer in Ghana (Incidence & mortality of Cervical Cancer)? 2. What are the interventions have been done in SA in recognition of the burden of cervical cancer? Are there any Strategic plans? Policies? (This will highlight any efforts which have been attempted to address the problem under study and outcomes of this effort) 3. Missed the gap (Why is the study being conducted?) |
| **Literature review** | 4. Did not follow specific objectives (literature review should be reorganized to capture what is currently known about the proposed study outcomes/objectives, and it should provide available evidence about the topic under study from a global perspective to local)    a. Missed literature on Cervical Cancer Uptake    b. Missed literature on barriers to Cervical Cancer uptake   c. Factors influencing Cervical Cancer uptake applying the health belief model |
| **Problem Statement** | 5. Problem under study is not stated clearly 6. Missed the knowledge gap (what information is lacking in the literature and what cannot be done because that information is not available? |
| **Justification** | 7. Missed the benefits of study findings (to whom, why and how will the study findings benefit the beneficiaries)  8. Who are the beneficiaries of these findings? |
| **Objectives** | 9. Objective number 2 is not clear “the study determines the association between sociodemographic characteristics and constructs under the lens of the health belief model”- misses the element of cervical cancer screening uptake. |
| **Methodology** | 1. Missed the study duration (only mentions study was conducted on “March 2019”- it is not clear if this is the beginning or the end of the study). 2. What was the reason of doing convenience sampling technique? 3. How the Health Belief Model was introduced during data collection is not clear. 4. What was the criteria to conduct questionnaire pre-test among 15 women in the pilot study? 5. Prevalence of Cervical Cancer Screening uptake is >10%, were the alternative to logistic regression considered? 6. In the data analysis section SPSS is not cited correctly 7. Study design should be changed to analytical cross-sectional design as logistic regression was done 8. Why was classical analysis (chi-square) done after the regression analysis and what was the reason to do chi-square and logistic regression as per the objectives? It is not clear what method answers what objective 9. Were the priori confounders considered? And how were they controlled for? 10. Overall data analysis section should be revised and reorganized and should state clearly which method answers what objectives. |
| **Results** | 1. It is important to mention the total sample size while reporting the results. 2. “N” showing total sample size should appear at the title of the table. 3. Results did not follow the specific objectives, makes it difficult to follow up. 4. What was being controlled for in the multivariable logistic regression analysis? Missed the bivariate analysis results 5. Overall results section should be re-organized per the specific objectives to enable the reader to follow through |
| **Discussion** | 1. Discussion is also per the specific objectives; we do not discuss the background characteristics of study participants (Objective 0) 2. When mentioning study similarities and differences it is worth mentioning study areas and the study populations for clarity 3. Discussion did not follow specific objectives, missed the link between the study findings and the possible explanations basing on policies and interventions in place regarding Cervical Cancer screening. |
| **Strengths and limitations** | 4. How is the current study able to improve Cervical Cancer screening uptake in Ghana? Please elaborate 5. How did the sampling design affect your study findings? Please elaborate more 6. What is the contribution of the current study to the scientific community? |
| **Conclusion** | 7. Recommendations are not clearly stated (to whom, and what actions should be done, with what resources and under what timeframe) 8. What is the way forward basing on the study findings? |
